# Book Reviews

**Published:** 1872-10-15

**Authors:** 


### Book Reviews.

The Physiology of Man. Designed to represent the existing state of Physiological Science, as applied to the functions of the human body. By Austin Flint, Jr., M.D. etc. Part IV. *Nervous System.* New York: D. Appleton & Co., Publishers.

This long looked-for and anxiously expected volume of Prof. Flint's Physiology is at length completed. It forms a volume of 461 pages, uniform with the previous numbers of the series. The physiological anatomy and the functions of the nervous system are the topics considered in this fourth part; the special senses being reserved for a fifth and last volume of the series. The author states that he has from the first looked upon this division of the work as the most difficult and the most important of all, and feels that this volume will be regarded with more critical interest than any one of the series; and if he has succeeded, even in a measure, in giving in it a satisfactory representation of our present positive knowledge, he considers that no apology is necessary for the length of time occupied in its preparation. For two and a half years he has been almost unremittingly engaged in writing this volume, and has endeavored to overcome, rather than avoid the difficulties which have presented themselves in the investigation of important questions, which are as yet regarded by many as unsettled. "Adhering conscientiously to the positive method of study, the author has endeavored to present an account of the nervous system, which, though it will undoubtedly be extended by future investigation, is made up mainly of statements of facts that will probably not undergo serious modification as we advance in our knowledge of the subject."

The author states that the fifth or last volume, which is to be devoted to the special senses and generation, is so far advanced that it is hoped that the entire work will be finished within a year.

The publishers of this series, with a view to presenting a complete work on the physiology and pathology of the nervous system, are about to issue this present volume of Prof. Flint's in connection with the lately issued work on Nervous Diseases by Prof. Hammond.

On the Functional Diseases of the Renal, Urinary, and Reproductive Organs, with a general review of Urinary Pathology. By D. Campbell Black, M. D., L. R. C. S. Lindsay & Blakiston, Philadelphia.

This is a small volume of 286 pages, and includes a concise, pratical consideration of the following topics: The conditions that affect the secretion of urine, with special reference to suppression. Retention of urine; its varieties, causes and treatment. Irritable bladder; strangury. Pathology and treatment of nocturnal enuresis, and spermatic incontinence. Sterilty in the male. Male impotency. Anomalous urethral discharges.

The Treatment of Syphilis, with Subcutaneous Sublimate Injections, by Dr. George Lewin, Berlin. Translated by Carl Prœgler, M.D., and E. H. Gale, M. D. Philadelphia: Lindsay & Blakiston.

The author states, in his preface, that nearly seven years have elapsed, since he introduced the subcutaneous sublimate injection into the syphilitic wards under his charge, in the Royal Charite Hospital, Berlin, and that during that time he has treated by this new method upwards of 2,000 patients. The special advantages claimed for it, are the quickness and precision of the results obtained, and the small per centage of relapses that occur. This method has the advantage, he says, over other anti-syphilitic cures, inasmuch as it requires no preparation of the system for, nor any after-cure in consequence of the medicine taken, because we can proceed at once *in medias res*, without losing any time whatever.

The instrument used in the operation is a special syringe, modified by Dr. Lewin, and manufactured by Geo. Tieman & Co., New York. In private practice, each patient must have his own needle, so as to prevent all possibility of infection. "The operation itself is the same as with any other subcutaneous injection. The cuticle should be raised, and the point of the syringe pushed into the middle of the cellular tissue, thus elevated. Be careful and not penerate so deeply as to injure the muscle; neither go too superficially, because the medicated fluid, remaining in the tense and unyielding stratum of cuticle, will not be absorbed, but give rise to inflammation and sloughing of the part; therefore, it is of vital importance that the medicated fluid be deposited in the meshes of the cellular tissue." The author uses three different strengths of solution, one of three grains to the ounce, one of four, and one of six grains to the ounce. "More concentrated solutions than these, cause often intense local inflammation and sensitiveness, and the formation of abscesses. Which of the solutions to use, in any particular case, must be determined by idiosyncrasy of the patient, sensitiveness, susceptibility, etc. Generally I prefer in practice, the middle strong solution, and with patients in Charite Hospital, I use it mostly." The partial dose for each injection, he states to be from $\frac{1}{10}$ to $\frac{1}{8}$ of a grain; the injections being repeated from one to three or four times daily, according to circumstances, severity of symptoms, etc.

The extended table of statistics given, as well as the detailed record of cases, seems to fully confirm the theoretical advantages claimed for this form of treatment, and we recommend the work to the attention of those interested in this department.

